# Progress in Surgery

**Published:** 1898-06-04

**Authors:** 


### Progress in Surgery.

### GENERAL SURGERY.

(*Continued from page* 150.)

**Fractures and Dislocations: Upper Extremity.**—The retention of the articular surfaces in position after reduction of acromio-clavicular dislocation is always a matter of some difficulty. The good result obtained in a case of this kind reported by T. D. Rhoads,[17] and the simplicity of the application, would tend to recommend the following method, described by him as a suitable one in the treatment of this refractory lesion. A wedge-shaped pad of absorbent cotton rolled in a towel is placed under the arm, the apex of the pad being pressed firmly into the axilla. A folded towel is placed across the shoulder so as to exert pressure over a broad area at the site of injury, and a strap two inches wide is thrown across the shoulder and under the elbow (which is protected by a pad of cotton-wool), and tightened as much as the patient can well bear, the point where pressure is exerted being internal to the joint. The scapula, clavicle, and trapezius muscle are thus controlled, without causing, the pain of pressure directly over the site of injury. A simple bandage passed under the opposite axilla prevents the strap from slipping off the shoulder. The placing of the wedge-shaped pad under the arm, with the broad base downwards, makes it possible to exert pressure on the arm upwards, outwards, and backwards, raising the glenoid cavity and scapula, while the clavicle is pushed downwards by the same force and thus prevented from again riding up over the acromio. A roller bandage round the chest keeps the arm and elbow to the side. The apparatus devised by T. R. Marshall[18] for the treatment of this dislocation in his own case is also at once simple and effectual. It can be made at home or by a saddler. It is composed of a pad three-quarters of an inch in thickness, covering well the upper aspect of the shoulder, and extending above from the root of the neck down over the shoulder to the arm four inches below the axilla. Here it is secured to the arm by a strap sewed to its lower end. The upper end is held in position by a strap passing across the chest under the opposite axilla. Local pressure over the acromil end of the clavicle is made by a third strap, two inches wide, which passes over it round the shoulder and underneath the axilla, which is protected by a pad from pressure. The strap is secured to the shoulder pad by two transverse strips. If necessary, immediately after the injury, the arm may be held securely to the side by two adjustable straps, extending from the end of the pad on the arm to the chest strap as it passes underneath the opposite axilla. The forearm should then be semiflexed and placed in a sling. Charles L. Scudder[19] describes two cases of dislocation of the shoulder-joint in infants. One was, probably, a true congenital dislocation or misplacement of the joint, and the other a traumatic subspinous dislocation occurring at birth. In the former case the X-ray picture displayed an absence of bone about the region of the glenoid and the small size of the humeral epiphysis. E. R. Corson[20] has shown that in severe cases of Colles's fracture, besides the fracture of the lower end of the radius, whether simply transverse or comminuted, its styloid process is broken off, and with it the styloid process of the ulna, and that the two radio-ulnar ligaments and the internal lateral ligament are also ruptured. He treats such cases in the prone position on a light flat splint, with chief regard to the ruptured ligaments. The wrist and forearm are laid prone in a straight line on the splint, slightly padded, and extending from an inch below the elbow to the metacarpo-phalangeal joints. To overcome the displacement of the ulna towards the palmar surface, a wedge is made of a narrow roller bandage, sufficiently thick to bring the ulna into its natural place, and long enough to extend well over the fusiform bone, thus bridging the ulno-carpal joint and forming a *point d'appui* for the retaining bandage. A bandage one and a half inches wide, starting on the ulnar side over the pad is carried round under the splint to the radial side, and over the dorsum of the wrist to the point of starting, making two turns; then from the radial side the bandage is carried over the dorsum of the hand to the metacarpo-phalangeal joint of the little finger, under the palm to the corresponding joint of the index finger, and finally across the dorsum of the hand diagonally to the point of starting, thus making a figure-of-eight; and this is repeated two or three times. The bandage is then continued up the forearm to the upper end of the splint. This keeps the hand in a straight line with the forearm. The bandage is kept on six weeks, the parts being gently massaged several times daily. In backward dislocation of the first thumb phalanx, J. Hutchinson, jun.,[21] advocates a careful trial being first given to the manipulation method described so well by M. Farabeuf and others. If this fails a tenotome should be introduced from the dorsum behind the projecting base of the phalanx, so as to divide the glenoid ligament and allow the sesamoid bones to slip on either side of the metacarpal head. This method of section, which dates back as far as the time of Desault, divides no important structures, is perfectly safe if aseptic precautions be observed, and will not interfere with the subsequent utility of the joint. It is, therefore, preferable to all methods involving a palmar incision.

**Lower Extremity.**—The literature of the subject of spontaneous dislocation of the hip-joint, occurring during the course of the acute infectious diseases, is carefully set out by D. N. Eisendrath.[22] Spontaneous dislocation may occur during typhoid fever, scarlatina, and acute articular rheumatism, especially in the first-named. No cases have ever been reported occurring during measles. Franke, in a recent article upon diseased bones and joints in influenza, reports two cases in which there was severe pain and exudation in the ankle and knee joints. Since influenza must be classed as an acute infectious disease, it seems very plausible, according to Eisendrath, that after exudation into the hip-joint, which probably existed in a case reported by him, dislocation may be the result of an attack of influenza. Some practical points upon fracture of the thigh-bone in babies is given by R. H. M. Dawbarn.[23] Amongst other methods of treatment he mentions the device of flexing the baby's thigh sharply against its abdomen, and retaining it there by aid of bandaging, pasteboard splints, or adhesive plaster, short co-optation splints of some kind being also employed. If shortening be absent or insignificant in amount in fracture, either of neck or shaft, in infants, this plan has obvious advantages; and especially so when the fracture is high in the shaft, with consequent angular deformity. He says that Van Arsdale's method with a pasteboard triangle (recently described at the American Medical Association in Philadelphia) is an exceptionally neat and effective way of splinting. As a matter of routine, and for some weeks, he gives zinc phosphide in small doses. In a case of constantly recurring sublaxation of the knee-joint, due to extreme laxity of the joint structures, A. H. Butcher[24] successfully produced a stiff knee by the following operation: by means of a slightly incurved incision across the part of the joint and division of the ligamentum patellæ, crucial, and lateral ligaments, the semi-lunar cartilages were removed, together with the articular cartilage from the femur, tibia, and patellæ. The tuberosities of the tibia were slightly hollowed out to receive the condyles of the femur. The tibia and femur were afterwards secured in position by two ivory pegs. Mr. Butcher was of opinion that the result was far preferable to any that could have been obtained by the ordinary method of incision. In the open method of suture of old fractures of the patella A. E. Barker[25] has recently adopted the following plan in five or six cases: A curved incision is made, with the convexity upwards, at the level of the upper fragment, and by means of the knife the fibrous tissue uniting the fractured surfaces and the remains of the patella bursa are cleared from between the fragments, and left attached to the deep surface of the skin flap. The bones are then brought together by a silver suture passed behind the fragments, in the same manner that this surgeon does in subcutaneous suture of the patella in recent fractures. The result in his cases was more satisfactory than in those operated on by other methods. In a long article on "When Should a Transverse Fracture of the Patella be Treated by Wiring?" Payton T. B. Beale[26] discusses the various conditions in which this operation may be performed. The operation should, he says, be performed immediately after the fracture has occurred only when the two following conditions can be observed together, viz., that blood has not begun to be effused, and that no direct violence has been applied to the part. It is, therefore, clear that in the great majority of cases which are otherwise suitable for wiring, the operation should not be performed for some time after the fracture has occurred. The age and occupation of the patient are also points to be considered. H. Lilienthal[27] reports another instance of fractured patella treated successfully by massage, so that the patient had been out of bed and walking eight days after the accident. Massage, he was sure, had a two-fold advantage, viz., (1) the getting rid of the effusion, and (2) the passive motion produced at the same time. J. Lynn Thomas[28] produces a skiagraph of a case of the very rare dislocation of the body of the astragalus backwards. There is a fracture through the neck of the astragalus, the body is dislocated backwards, the trochlear surface being in contact with the tendo-achillis, there being an axial rotation of 90 deg. backwards through its transverse axis. There is no formation of bone in the gap between the neck and displaced body of the astragalus, for the fibular malleolus is distinctly seen. The tibial malleolus is seen resting upon the two segments.

[17] Annals of Surgery, Jan., 1898, p. 40. [18] New York Med. Rec., Jan. 22, 1898, p. 143. [19] Amer. Jour. Med. Sci., Feb., 1898, p. 125. [20] New York Med. Rec, Jan. 15, 1898, p. 82. [21] Brit. Med. Jour., Jan. 15, 1898, p. 189. [22] Annals of Surgery, Oct., 1897, p. 448. [23] Ibid., p. 480. [24] Brit. Med. Jour., Dec., 1897. [25] Lancet, April 2, 1898, p. 922. [26] Treatment, Jan. 27, 1898. [27] New York Med. Rec., Feb. 5, 1898, p. 203. [28] Annals of Surgery, 1897, p. 704.

